# Tuberculosis Susceptibility and Vaccine Protection Are Independently Controlled by Host Genotype

**DOI:** 10.1128/mBio.01516-16

**Published:** 2016-09-20

**Authors:** Clare M. Smith, Megan K. Proulx, Andrew J. Olive, Dominick Laddy, Bibhuti B. Mishra, Caitlin Moss, Nuria Martinez Gutierrez, Michelle M. Bellerose, Palmira Barreira-Silva, Jia Yao Phuah, Richard E. Baker, Samuel M. Behar, Hardy Kornfeld, Thomas G. Evans, Gillian Beamer, Christopher M. Sassetti

**Affiliations:** a Department of Microbiology and Physiological Systems, University of Massachusetts Medical School, Worcester, Massachusetts, USA; b AERAS, Rockville, Maryland, USA; c Department of Medicine, University of Massachusetts Medical School, Worcester, Massachusetts, USA; d Department of Infectious Diseases and Global Health, Cummings School of Veterinary Medicine, Tufts University, North Grafton, Massachusetts, USA; e Life and Health Sciences Research Institute, School of Health Sciences, University of Minho, Braga, Portugal; f ICVS/3B's, Portuguese Government Associate Laboratory, Braga/Guimaraes, Portugal

## Abstract

The outcome of *Mycobacterium tuberculosis* infection and the immunological response to the bacillus Calmette-Guerin (BCG) vaccine are highly variable in humans. Deciphering the relative importance of host genetics, environment, and vaccine preparation for the efficacy of BCG has proven difficult in natural populations. We developed a model system that captures the breadth of immunological responses observed in outbred individual mice, which can be used to understand the contribution of host genetics to vaccine efficacy. This system employs a panel of highly diverse inbred mouse strains, consisting of the founders and recombinant progeny of the “Collaborative Cross” project. Unlike natural populations, the structure of this panel allows the serial evaluation of genetically identical individuals and the quantification of genotype-specific effects of interventions such as vaccination. When analyzed in the aggregate, our panel resembled natural populations in several important respects: the animals displayed a broad range of susceptibility to *M. tuberculosis*, differed in their immunological responses to infection, and were not durably protected by BCG vaccination. However, when analyzed at the genotype level, we found that these phenotypic differences were heritable. *M. tuberculosis* susceptibility varied between lines, from extreme sensitivity to progressive *M. tuberculosis* clearance. Similarly, only a minority of the genotypes was protected by vaccination. The efficacy of BCG was genetically separable from susceptibility to *M. tuberculosis*, and the lack of efficacy in the aggregate analysis was driven by nonresponsive lines that mounted a qualitatively distinct response to infection. These observations support an important role for host genetic diversity in determining BCG efficacy and provide a new resource to rationally develop more broadly efficacious vaccines.

## IMPORTANCE

Tuberculosis (TB) remains an urgent global health crisis, and the efficacy of the currently used TB vaccine, *M. bovis* BCG, is highly variable. The design of more broadly efficacious vaccines depends on understanding the factors that limit the protection imparted by BCG. While these complex factors are difficult to disentangle in natural populations, we used a model population of mice to understand the role of host genetic composition in BCG efficacy. We found that the ability of BCG to protect mice with different genotypes was remarkably variable. The efficacy of BCG did not depend on the intrinsic susceptibility of the animal but, instead, correlated with qualitative differences in the immune responses to the pathogen. These studies suggest that host genetic polymorphism is a critical determinant of vaccine efficacy and provide a model system to develop interventions that will be useful in genetically diverse populations.

## INTRODUCTION

The outcome of an encounter with *Mycobacterium tuberculosis* is highly variable. Most individuals contain the infection and can remain asymptomatic for a lifetime. Only a fraction of infected individuals develop active disease, and even among these, the timing, location, and presentation of the pathology is remarkably diverse (1). The underlying basis of the variable outcomes of *M. tuberculosis* infection is unknown and likely involves a complex interplay between environmental factors and genetic variation in both host and pathogen (2). Classic evidence for a role of host genetics driving disease outcome comes from twin studies showing a higher tuberculosis (TB) concordance rate in monozygotic than in dizygotic twins (3, 4). More recently, linkage analyses defined rare Mendelian traits that cause extreme susceptibility to mycobacterial disease in children (5–9), and a variety of case-control (10, 11), linkage (12), or genome-wide association studies (13, 14) have implicated more common genetic variants in TB risk. The identification of these TB-associated polymorphisms provides valuable insight into the pathogenesis of this disease, as many of the identified genes function in the establishment of a protective Th1-biased cell-mediated immune response (15), regulate disease-promoting inflammation (16, 17), or alter the pathogen’s intracellular environment (18). However, these known mechanisms explain only a small fraction of the variability observed in natural populations (2), suggesting an important role for interactions between these and other disease-modifying polymorphisms.

This diversity in TB susceptibility is mirrored in the variable efficacy of vaccination for this disease. The only TB vaccine that has been shown to protect humans is an attenuated strain of *Mycobacterium bovis*, bacillus Calmette-Geurin (BCG). This vaccine has been administered to more than 3 billion humans since it was developed in the 1920s. In the subsequent decades, studies in different geographic regions and ethnic populations have produced widely variable estimates of BCG’s effect. In several populations, the efficacy of BCG is estimated to be greater than 75%. However, in regions where TB remains endemic, no significant protection from pulmonary TB in adults can be detected (19, 20). This variable efficacy could be due to differences in the vaccine strain or preparation, the environment, or the genetic background of the host. The roles of genetic variation in the vaccine strain (21–24) and previous exposure to environmental mycobacteria (25–27) have been investigated extensively. In contrast, the role of host genetic variation in BCG efficacy has been more difficult to quantify. It is clear that variations in many immune mediators are highly heritable (28–30). Many of these heritable variations affect mediators that are likely to be relevant to *M. tuberculosis* immunity, such as the number of central memory T cells or the abundance of cytokines like interleukin 12p40 (IL-12p40), granulocyte-macrophage colony-stimulating factor (GM-CSF), alpha interferon (IFN-α), and IL-6 (31). Indeed, numerous studies suggest that the immunological response to mycobacterial infection (32–35) or BCG vaccination (36, 37) is heritable. However, the relationship between these immunological markers and vaccine efficacy is unknown and very difficult to address in natural populations. Thus, while there is reason to suspect that BCG efficacy is influenced by genetic variation, it has proven difficult to dissociate these effects from other confounding variables. In particular, the effect of BCG is difficult to dissociate from the intrinsic TB susceptibility of each individual in a natural population.

In theory, animal models could be used to dissect the role of genetic diversity in vaccine protection. However, while the mouse model of TB has been very useful for understanding the mechanisms underlying Mendelian susceptibility to TB, this approach has proven less useful for understanding the complex trait genetics that that have been shown to underlie TB susceptibility in mice, with only 2 host loci so far identified from forward-genetic approaches (38–40). A fundamental limitation of the classic inbred strains of *Mus musculus domesticus* that are generally used to model TB is their genetic homogeneity, as 90 to 95% of these animals’ genomes are estimated to be functionally identical (41). As a result, these mouse strains mount qualitatively comparable immune responses to this infection and vary only modestly in their susceptibility to *M. tuberculosis* (42). In virtually every strain, *M. tuberculosis* grows logarithmically for 2 to 3 weeks, at which point bacterial growth is restricted by a strong Th1-biased CD4^+^ T cell response. Immunity depends largely on CD4^+^ T cell-derived IFN-γ and allows the animal to survive for several months harboring a relatively constant burden of bacteria. BCG vaccination also produces a relatively homogenous effect in a variety of standard laboratory strains of mice (43–45), accelerating the initiation of adaptive immunity by several days, reducing the peak burden of bacteria by approximately 10-fold (46), and extending survival (47). It is often noted that the mouse model does not reproduce many of the aspects of TB disease seen in humans or nonhuman primates, including variable histopathology (48), progressive bacterial killing (49), and widely varying susceptibility (e.g., see reference 50). While these discrepancies are generally attributed to species difference, the lack of variation observed in mice could also reflect the low genetic diversity between classical inbred strains (51). Thus, the true range of TB-related traits that can be modeled in mice remains unclear.

Tractable model systems that incorporate relevant genetic diversity could be used to decipher the genetic determinants of both TB susceptibility and vaccine protection. Recently, a number of related outbred model mouse populations have been developed based on the same set of eight genetically diverse founder lines, which represent all three *M. musculus* subspecies. The outbred progeny of these strains, called the Diversity Outbred (DO) population, represent genetic diversity similar to that of a human outbred population (52, 53) and display a remarkable heterogeneity in *M. tuberculosis* susceptibility (54). However, the completely outbred structure of this population imposes several limitations on the resource. Most notably, each genotype is represented by only a single animal. As a result, it is difficult to quantify the genotype-specific effect of an intervention, such as vaccination. To complement this resource, a panel of recombinant inbred lines derived from the same founders was generated and is called the “Collaborative Cross” (CC) (55, 56). The CC panel retains the genetic diversity of DO animals but allows each genotype to be infinitely reproduced. We took advantage of the unique structure of the CC population to investigate the relationship between host genotype, TB susceptibility, and BCG efficacy. We found that the panel of parental and recombinant CC strains (hereinafter named the “diversity panel”) reproduced many aspects of outbred populations and encompassed reproducible phenotypes that extend well beyond those observed in standard inbred strains. Using the diversity panel, we found that the efficacy of BCG is genetically dissociable from susceptibility to TB and correlates with intrinsic immune biases in the strains. This new genetic resource identified an important role for host genetic diversity in vaccine efficacy and provides new approaches to understand the immunological basis of protection and to optimize vaccines for outbred populations.

## RESULTS

### Genetically diverse mice display a broad range of phenotypes upon *M. tuberculosis* infection.

In order to independently assess the contribution of host genotype to *M. tuberculosis* susceptibility and vaccine protection, we assembled a population of highly diverse and reproducible inbred lines. This diversity panel contained the eight founder lines of the CC and DO populations, which include five relatively diverse classical inbred strains (A/J, C57BL/6J, 129S1/SvImJ, NOD/LtJ, and NZO/H1LtJ) and three wild-derived inbred strains (CAST/EiJ, PWK/PhJ, and WSB/EiJ), hereinafter abbreviated to A/J, B6, 129, NOD, NZO, CAST, PWK, and WSB. We also took advantage of additional recombinant CC lines, since the range observed for polygenic traits in this type of panel is often much greater in the genetically mosaic offspring than in the founders (57, 58). Recombinant lines were included in this panel based on their relative resistance or susceptibility to *M. tuberculosis* after intravenous challenge, which was determined in a related study.

**FIG 1:**
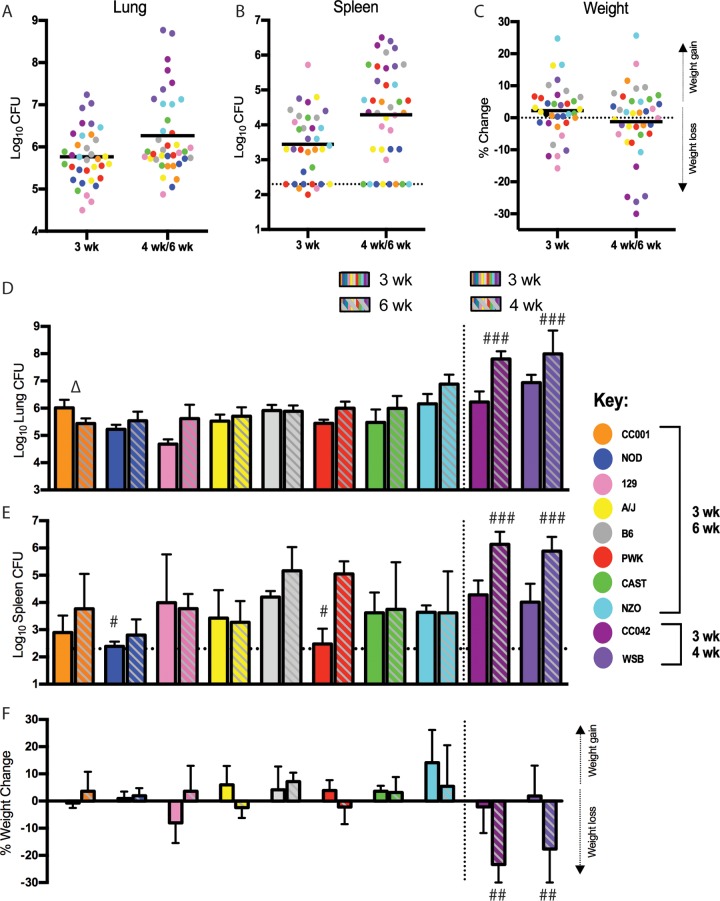
*M. tuberculosis* disease phenotypes in diverse mice. (A to C) Lung CFU (A), spleen CFU (B), and weight change relative to initial weight (C) for individual mice, colored by genotype, at weeks 3 and 6 (for CC001, NOD, 129, A/J, B6, PWK, CAST, and NZO lines) or weeks 3 and 4 (for WSB and CC042 lines, which were moribund by week 4). (D to F) Average lung CFU (D), average spleen CFU (E), and average weight change (F) broken out by mouse genotype. (D to F) All data are the average value ± SD for 3 or 4 mice per strain at each time point. A statistically significant comparison within mouse strains (*P* < 0.05) using Student’s *t* test is indicated by a triangle (▵), and hash symbols (#) indicate statistical significance compared to the result for B6 mice via one-way ANOVA with Tukey’s multiple comparison test (#, *P* < 0.05; ##, *P* < 0.01; ###, *P* < 0.001)

To characterize the range of *M. tuberculosis* susceptibility represented in the diversity panel, we infected groups of each genotype with strain H37Rv via the aerosol route and monitored disease over time. When the population was analyzed in the aggregate (Fig. 1A, B, and C), we observed a range of susceptibility similar to that observed in completely outbred DO animals derived from the same founders (54). The lung and spleen CFU burdens varied over a range of 1,000- to 10,000-fold, and some animals required euthanasia after only 4 weeks of infection. When the panel was assessed at the genotype level (Fig. 1D, E, and F), we found that these differences in susceptibility were reproducible within each genotype and were therefore highly heritable. Based on the bacterial burdens in lungs at 6 weeks postinfection, the genotypes could be ranked based on susceptibility (Fig. 1D). The B6 strain, generally considered to be the most resistant of the classical inbred lines (59), showed intermediate susceptibility in our panel, maintaining lung pathogen burdens of approximately 10^6^ CFU throughout the infection. The CC recombinant line, CC001, proved to be the most resistant to infection by 6 weeks, while the wild-derived line of *M. musculus domesticus*, WSB, was the most susceptible. The phenotypes of these outlier strains were notable. CC001 was the only line in the panel in which the lung CFU burdens decreased between weeks 3 and 6 postinfection (*P* = 0.044 by *t* test). This relatively modest decrease was the first indication that this strain progressively kills *M. tuberculosis* over time (as shown in the subsequent studies presented below; see Fig. S2 in the supplemental material). In contrast, the WSB and CC042 strains were both unable to control bacterial replication (*P* = 0.0001 for WSB and *P* < 0.0001 for CC042 compared to B6 via one-way analysis of variance [ANOVA] with Tukey’s multiple comparison test), lost weight, (*P* = 0.0039 for WSB and *P* = 0.0004 for CC042 compared to B6 via one-way ANOVA with Tukey’s multiple comparison test), and were moribund after only 4 weeks of infection. Thus, these genotypes were as susceptible as knockout mice lacking critical aspects of CD4^+^ T cell-driven immunity, such as inducible nitric oxide synthase (NOS2) (60) or major histocompatibility complex class II (MHC-II) (61).

The ability to accurately measure multiple metrics of disease over time in reproducible lines allowed the identification of traits that are genetically separable. For example, the rate at which bacteria disseminate from the lung to the spleen varied across the panel. Despite harboring similar numbers of *M. tuberculosis* bacteria in the lungs at 3 weeks postinfection, NOD mice and the wild-derived strain of *M. musculus musculus*, PWK, harbored significantly fewer CFU in their spleens than B6 (*P* = 0.020 for NOD versus B6 and *P* = 0.028 for PWK versus B6 by one-way ANOVA with Tukey’s multiple comparison test). In the spleens of PWK mice, the *M. tuberculosis* burden increased between 3 and 6 weeks postinfection, indicating that *M. tuberculosis* is able to replicate at this site. Thus, the early deficit in spleen CFU in this genotype could reflect delayed dissemination from the lung, which occurs during this period and has previously been shown to vary with host genotype (62). We conclude that the diversity panel encompasses a wide variation in TB susceptibility traits and that different aspects of disease may be controlled by distinct genetic polymorphisms.

### TB pathogenesis and immune response differ qualitatively between strains.

**FIG 2:**
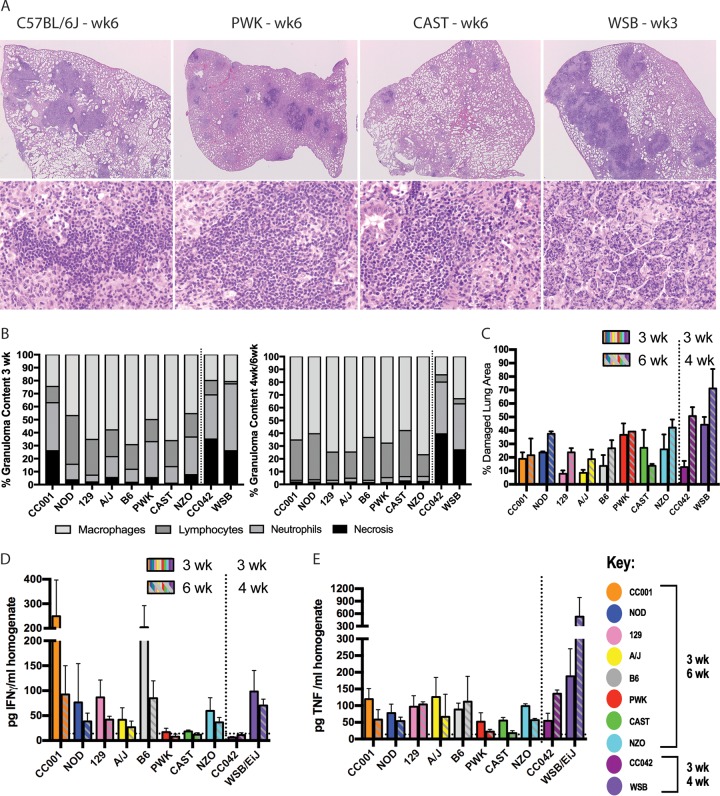
Distinct cytokine environments and histopathology results in diverse mice. (A) Representative images from B6, PWK, CAST, and WSB mice at ×2 and ×20 magnification. (B) Proportional areas of granuloma occupied by necrosis, neutrophils, lymphocytes, and macrophages at 3 weeks postinfection (left) and 4 weeks or 6 weeks postinfection (right). Bar heights represent the average values from 10 randomly selected granulomas for each of 3 to 4 mice per mouse strain. (C) Percentage of lung area that was damaged, measured using ImageJ to trace lesion size relative to the whole lung section. Bar heights represent the average values from 3 or 4 mice per strain. Error bars show standard deviations. (D and E) IFN-γ (D) and TNF (E) cytokines measured in homogenates of infected lungs at 3 and 6 weeks (or 3 and 4 weeks for WSB and CC042 mice) postinfection. All data are the average value and SD from 3 or 4 mice per strain at each time point, assayed in technical duplicate. Limit of detection (LOD) was calculated as twofold the background (17 pg/ml).

The polygenic basis of susceptibility in the diversity panel suggested that distinct inflammatory and/or immunological pathways might underlie disease outcomes. Indeed, the lungs of infected mice displayed lesional heterogeneity between strains over time. At 3 and 6 weeks of *M. tuberculosis* infection, the lungs of B6 mice (the standard for mouse *M. tuberculosis* studies) contained typical lesions for this strain, i.e., multifocal coalescing histiocytic alveolar pneumonia with perivascular and peribronchiolar lymphocytic aggregates, no necrosis, and diminishing neutrophils over time (Fig. 2A to C). The lungs of several similarly resistant inbred strains (PWK and CAST) shared these characteristics. In contrast, after only 3 weeks of infection, the susceptible WSB strain had already developed dense neutrophilic inflammatory infiltrates in small and large airways, which after one additional week of infection progressed to widespread necrosis of inflammatory cells and lung tissue, associated with morbidity. Interestingly, the *M. tuberculosis* burden alone did not predict the extent of lung damage or the type of microscopic lesions in all cases. For example, the lungs of CC001 mice displayed early neutrophil recruitment and necrosis that was not apparent in other similarly resistant lines and was resolved by 6 weeks postinfection, when the bacterial burden had decreased in this strain. Thus, the CC001 strain has some capacity to tolerate and resolve early necrosis and neutrophilic-mediated lung damage due to *M. tuberculosis* infection.

Qualitative differences in the immune response were more apparent when cytokine levels were measured in the lungs (Fig. 2D and E). Immunity to *M. tuberculosis* in B6 mice depends on IFN-γ and tumor necrosis factor (TNF), which contribute to the effector function of CD4^+^ T cells (63, 64). Lung TNF levels were relatively consistent across the panel at 3 weeks postinfection and were only elevated in the moribund WSB mice after 4 weeks. In contrast, the IFN-γ levels in mice with these genotypes varied by nearly 20-fold at the early time point. The wild-derived lines PWK and CAST and the recombinant CC042 line expressed remarkably low levels of this cytokine that were at or below the limit of detection (LOD) of the assay (LOD = 17 pg/ml). Splenocytes from PWK and CAST mice both produced IFN-γ upon polyclonal stimulation *ex vivo* (see Fig. S1 in the supplemental material), indicating that these cells were capable of producing detectable levels of cytokine in our assay. Thus, the lack of IFN-γ expression in the lungs of PWK, CAST, and CC042 mice was not due to an inherent inability to express the cytokine. Instead, these lines appear to mount a distinct response to *M. tuberculosis* infection, and the relative levels of resistance of the CAST and PWK lines indicate that these responses can be effective in the absence of high levels of IFN-γ.

### Host genotype determines BCG efficacy.

To determine whether genetic polymorphism could influence the degree of protection conferred by vaccination, we immunized a panel of diverse mouse strains with BCG by the subcutaneous route. The mice used for the vaccination study consisted of the same CC founder strains, the highly resistant CC001 recombinant line, and two additional recombinant lines representing susceptible (CC019) and resistant (CC002) phenotypes. Twelve weeks after vaccination, viable BCG was not detected in spleen homogenates of all genotypes (LOD = 20 CFU/spleen), indicating that all mice were able to control infection with this attenuated strain. The mice were then challenged with *M. tuberculosis*, and the protection elicited by vaccination was determined at 4 and 14 weeks postinfection.

**FIG 3:**
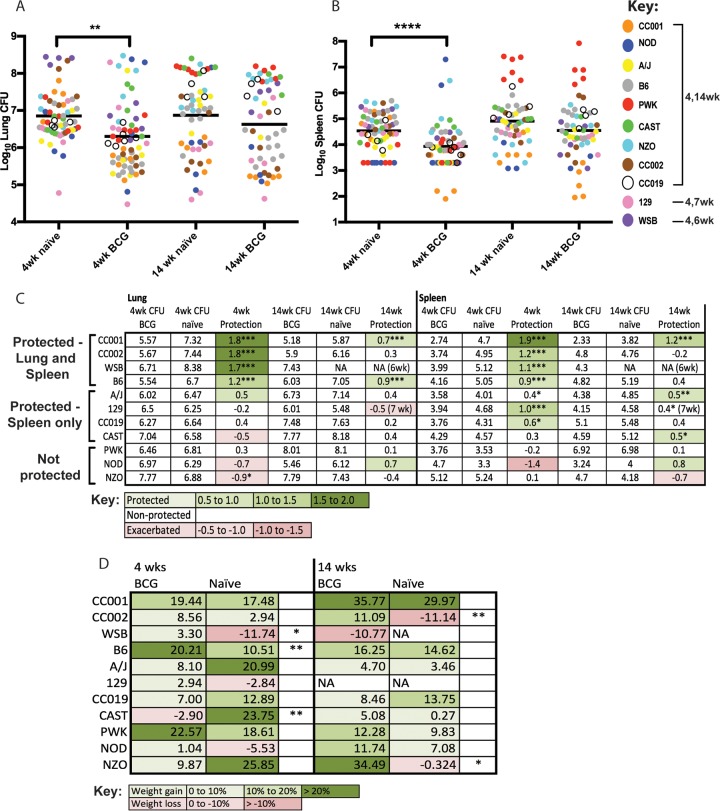
The effect of genetic background on BCG protection in diverse mice. (A and B) Lung CFU (A) and spleen CFU (B) in naive and vaccinated mice for all genotypes. Each point represents the CFU for an individual mouse at the indicated time point. (C) Fold protection of each mouse strain was calculated from the average CFU of naive compared to BCG-vaccinated groups at 4 and 14 weeks postinfection in the lung and spleen. Coloring indicates CFU reduction (green) or exacerbation (red) in the BCG-vaccinated group. “Protection” is defined as a statistically significant 0.5 log_10_ reduction in the BGC-vaccinated group. (D) Average percent weight change of each mouse strain relative to initial body weight for BCG-vaccinated and naive mice at 4 and 14 weeks. The percent weight changes were compared between naive and BCG-vaccinated mice within each mouse strain (unpaired *t* test). *n* = 6 mice per strain per time point for naive or BCG-vaccinated mice. *, *P* < 0.05; **, *P* < 0.01; ***, *P* < 0.001.

We initially analyzed this large panel as an aggregate population with 53 to 66 animals per group. As seen previously, the bacterial burdens in the lungs and spleens of these animals varied widely, and vaccination did not alter this variation (Fig. 3A and B). BCG vaccination reduced the mean *M. tuberculosis* burdens in the lungs or spleens by 1.7- to 4-fold at each time point, but even with this large group size, the BCG-mediated reduction of bacterial burden was only statistically significant at 4 weeks postinfection. Thus, no durable protection from *M. tuberculosis* growth could be detected in the aggregated data, which mirrors the lack of protection conferred by BCG in many natural outbred populations.

When the data were analyzed at the genotype level, it became clear that the variation in CFU burdens was driven by genotype-specific effects on both *M. tuberculosis* susceptibility and vaccine efficacy (Fig. 3C and D). Using the reduction in *M. tuberculosis* burden as a metric of vaccine efficacy, we defined “protection” as a decrease in CFU of greater than 0.5 log_10_ that reached statistical significance. Based on these criteria, only a subset of genotypes was protected by vaccination. Consistent with previous literature, BCG reduced the bacterial loads in the lungs and spleens of B6 mice by approximately 10-fold. This response was shared in three other lines, two recombinants (CC001 and CC002) and the wild-derived WSB line. In these genotypes, BCG exposure reduced the *M. tuberculosis* loads by 10- to 100-fold. Notably, these were the only genotypes in which vaccination protected the lung. BCG conferred protection in the spleens but not the lungs of four additional lines (129, CC019, CAST, and A/J). The remaining genotypes were not protected by vaccination in either organ at any time point. In these nonresponding lines, BCG vaccination either had no effect on the *M. tuberculosis* burden or was associated with an increase in mean CFU, which reached statistical significance for the NZO genotype (*P* < 0.05 by *t* test). In most cases, an increased bacterial burden correlated with weight loss (Fig. 3D). In mice of the WSB, CC002, and B6 genotypes, BCG vaccination reduced the bacterial burdens and significantly reversed weight loss at one of the two time points. The effects of BCG on CFU and weight loss were discordant for mice of the NZO and CC001 genotypes, reflecting unique phenotypes that will be discussed below.

### TB susceptibility and BCG efficacy are genetically separable traits.

The ability to serially evaluate the same host genotype allowed multiple traits to be measured independently in the vaccinated and unvaccinated state. To investigate potential mechanisms that determine vaccine efficacy, we searched for correlates of BCG-mediated protection among the traits measured during primary infection. These traits included lung cytokine measurements as a metric of adaptive immunity, CFU as a measure of antimicrobial capacity, and weight loss as a surrogate for systemic disease.

**FIG 4:**
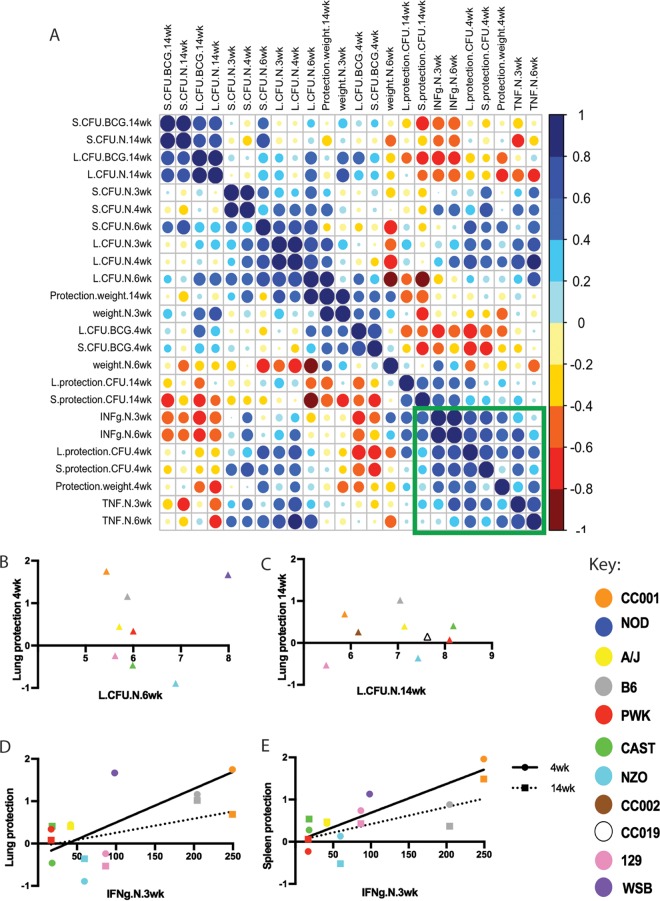
Phenotypic relationships between *M. tuberculosis* susceptibility and BCG efficacy. (A) Hierarchical clustering of correlations between metrics of susceptibility at weeks 3, 4, 6, and 14 postinfection and response to vaccination at weeks 4 and 14 postinfection. Blue indicates positive Pearson’s correlations, and red indicates negative correlations. “Protection” indicates the relative reduction in CFU or weight loss in the BCG-vaccinated group. “Weight” indicates percent weight change relative to initial body weight. “L” and “S” indicate lung and spleen, respectively; “N” and “BCG” indicate naive and vaccinated groups. Green box indicates the trait cluster containing metrics of BCG-mediated protection. (B and C) Lack of correlation between TB susceptibility and BCG efficacy at early (B) or late (C) time points. (D and E) Positive correlation between IFN-γ production after *M. tuberculosis* infection and BCG efficacy in the lung (D) or spleen (E). Solid lines are correlations of results at the 4-week time point (circles), and dotted lines are correlations of results at the 14-week time point (squares); each data point in panels B to E is the average value from 3 to 6 mice per genotype. Note that NOD mice are not displayed on the correlation plots due to the highly variable response of this strain (see Fig. S2J in the supplemental material).

Upon correlating disease and immune metrics with the degree of BCG protection, we identified a number of clusters of covariant traits. A single cluster contained virtually all traits associated with BCG-mediated protection, including all metrics of antimicrobial efficacy and protection from weight loss at 4 weeks postinfection (Fig. 4A, green box). The cluster did not contain any traits related to *M. tuberculosis* susceptibility in the unvaccinated state, and no specific correlation between *M. tuberculosis* susceptibility and BCG efficacy was evident (Fig. 4B and C). Furthermore, BCG caused the largest reductions in CFU in the CC001 and WSB lines, the most resistant and susceptible lines in the study, respectively (Fig. 4B, orange and purple). Thus, *M. tuberculosis* susceptibility and BCG efficacy were genetically separable.

In addition to all traits related to BCG protection, the BCG efficacy-related cluster of traits also included another metric of immune response that correlated strongly with the degree of BCG-mediated protection, the early production of IFN-γ by unvaccinated animals (Fig. 3D and E). The capacity to produce other T cell cytokines, such as TNF, was less predictive of BCG efficacy. Upon examination of individual genotypes, two of the wild-derived lines, CAST and PWK, were particularly notable for producing nearly undetectable levels of IFN-γ upon *M. tuberculosis* infection and receiving little or no benefit from BCG vaccination (Fig. 4D and E, red and green).

### Long-term protection depends on a combination of traits.

In this panel of genetically diverse mice, we found that the extent of disease at later time points depended on the intrinsic susceptibility of the animal as much as the effect of vaccination. For example, despite the nearly 100-fold reduction in lung *M. tuberculosis* burden at 4 weeks postinfection, BCG only marginally extended the survival of the highly susceptible WSB strain, from 4 to 6 weeks, and ultimately did not protect this strain from disease. Conversely, even without vaccination, the resistant CC001 and CC002 strains had the remarkable ability to kill 90 to 95% of *M. tuberculosis* bacteria between 4 and 14 weeks postinfection (Fig. 3; see also Fig S2 in the supplemental material). As a result, unvaccinated animals of these genotypes harbored bacterial burdens at the later time point that were similar to the burdens in the vaccinated B6 group. The relative resistance of this CC001 line likely explains its ability to gain weight throughout the infection, regardless of BCG vaccination (Fig. 3D).

**FIG 5:**
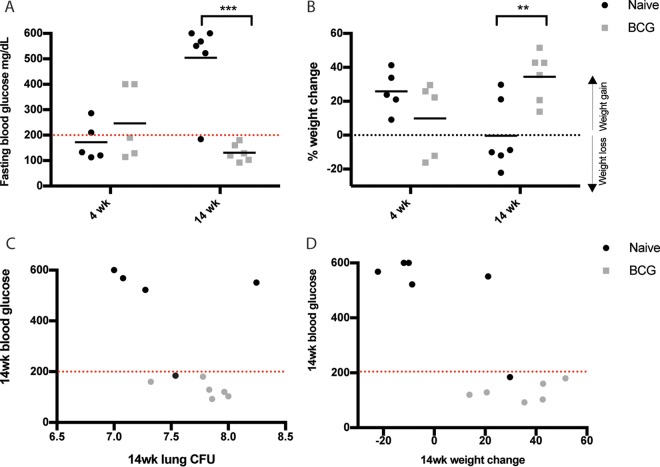
BCG protects *M. tuberculosis*-infected NZO mice from diabetes. (A) Blood glucose measurements obtained from naive and BCG-vaccinated NZO mice at 4 and 14 weeks postinfection. Mice were fasted for 6 h prior to obtaining blood glucose measurements. Mice were considered diabetic if they had a blood glucose concentration greater than 200 mg/dl. (B) Percent weight changes of naive and BCG-vaccinated mice relative to initial mouse weights at 4 and 14 weeks postinfection. Correlation of blood glucose versus lung CFU (C) and correlation of blood glucose versus weight change (D) at 14 weeks postinfection in naive and BCG-vaccinated mice. Each data point is the value for an individual NZO mouse at each specified time point. Naive and BCG-vaccinated groups were compared by Student’s *t* test. *, *P* < 0.05; **, *P* < 0.01; ***, *P* < 0.001.

We also found that mice could benefit from vaccination even if the *M. tuberculosis* burden was not reduced. For example, even though BCG-vaccinated NZO mice harbored significantly more *M. tuberculosis* bacteria in their lungs than their unvaccinated counterparts at 4 weeks postinfection, vaccination still reversed *M. tuberculosis*-induced weight loss between 4 and 14 weeks (Fig. 3D). The NZO mouse strain has been described to develop a polygenic form of type II diabetes at about the same age as the animals used in our study (65). As chronic hyperglycemia can increase *M. tuberculosis* susceptibility in mice (66, 67), we hypothesized that an interaction between vaccination and diabetes initiation might underlie the paradoxical effects of BCG on *M. tuberculosis* burden and weight loss. Indeed, while 5 of 6 unvaccinated animals were found to have fasting blood glucose levels above 500 mg/dl at 14 weeks postinfection, 0 of 6 vaccinated animals were diabetic (Fig. 5A and B). In the NZO genotype, diabetes strongly predicted weight loss but not CFU (Fig. 5C and D). Our diversity mouse panel also included NOD mice that develop autoimmune type I diabetes (68). No significant effect of BCG on either *M. tuberculosis* burden or blood glucose concentration was evident in this strain, possibly due to the variable penetrance of diabetes (see Fig. S2J in the supplemental material). Thus, the major protective effect of BCG in *M. tuberculosis*-infected NZO mice appears to be the prevention of type II diabetes. Taken together, our observations in the genetic diversity panel suggest that while the protective efficacy of BCG can be separated from TB susceptibility, the ultimate effect of vaccination on the outcome of infection is influenced by a variety of factors, including intrinsic susceptibility to TB and complex interactions with comorbidities, such as diabetes.

## DISCUSSION

Predicting TB risk and rationally designing more effective interventions will ultimately require understanding the mechanisms that control the outcome of *M. tuberculosis* infection in a genetically diverse population. An ideal experimental model to dissect these mechanisms would encompass relevant genetic diversity and allow the serial evaluation of genetically identical individuals. To create such a population, we took advantage of the Collaborative Cross project, which identified genetically diverse founder lines of mice and generated recombinant inbred animals in which this diversity was reassorted to produce more extreme phenotypes. Our studies using this panel extended the phenotypic diversity described in the species and allowed the contributions of host genetics to TB susceptibility and BCG efficacy to be assessed independently.

The course of *M. tuberculosis* infection in the standard “mouse model” (B6 inbred strain) is often contrasted with the diversity of TB-related disease that is apparent in other species. For example, while *M. tuberculosis* can replicate continually at some sites in the lungs of cynomolgus macaques, *M. tuberculosis* is efficiently killed in most of the granulomas (49). These studies are consistent with observations from human autopsies, which similarly suggest that *M. tuberculosis* is eradicated from many granuloma and only replicates in a minority of sites (69). In contrast, with the exception of a few susceptible substrains (54, 70), *M. tuberculosis* infection of standard laboratory strains of *M. musculus domesticus* follows a very similar course; *M. tuberculosis* replication can generally be controlled, but the mice are unable to clear the pathogen or even to significantly reduce the lung bacterial burden during the persistent phase of infection when the *M. tuberculosis* burden remains stable. Our studies suggest that this homogeneity in TB pathogenesis reflects the genetic homogeneity of these strains, but not the phenotypic diversity of the species. Of course, the mouse lines used in this study may not capture all diversity previously described in mouse substrains. For example, the C3HeB/FeJ substrain forms caseating lung lesions upon *M. tuberculosis* infection (70), which closely resemble those of humans. Determining whether any mouse lines in the CC panel recapitulate this phenotype will require additional infections studies in parallel with C3HeB/FeJ animals.

Phenotypes ranging from progressive killing of *M. tuberculosis* to extreme susceptibility were present in our panel and appear to be based on different underlying genetic mechanisms. The ability to kill *M. tuberculosis* during the persistent phase of infection (between 4 and 14 weeks) was found only in recombinant animals and not the founder lines, suggesting that this is a multigenic trait that depends on the proper assortment of founder alleles. In contrast, several extreme phenotypes were evident in the more divergent wild-derived lines. The WSB line of *M. musculus domesticus* was extremely susceptible to *M. tuberculosis*. This line has also been recently reported to be modestly susceptible to influenza A virus infection (71). However, these animals are otherwise healthy and able to control the replication and dissemination of BCG, suggesting a fairly specific defect in immunity to *M. tuberculosis*. While the CAST line of *M. musculus castaneus* and the PWK line of *M. musculus musculus* appeared similarly as susceptible to TB as many standard lines, their response to infection differed from those of other genotypes in several respects. The lungs of CAST mice displayed a paucity of inflammatory infiltrate relative to the levels in other mice that harbored similar bacterial burdens, a trait also reported for this genotype during influenza virus infection (71). PWK animals were able to delay the dissemination of *M. tuberculosis* from the lung to the spleen. Most strikingly, both CAST and PWK mice produced barely detectable levels of IFN-γ during infection. While this cytokine is critical for *M. tuberculosis* immunity in B6 mice, these strains were still able to control bacterial replication. CD4^+^ T cells have been shown to control *M. tuberculosis* growth through IFN-γ-independent mechanisms in several settings (72, 73), and Th17 cells have been specifically implicated in protection (74). Together, these observations suggest that these highly divergent mouse lines might preferentially depend on non-Th1-biased CD4^+^ T cell responses for *M. tuberculosis* immunity.

The structure of our mouse population allowed us to determine that BCG efficacy is controlled independently of TB susceptibility in naive animals. These observations suggest that even the most susceptible individuals can benefit from vaccination. However, the genetic basis of vaccine-conferred protection remains unclear. The animals in our panel encode diverse MHC haplotypes, raising the possibility that H2 polymorphism could influence the degree of protection (40). However, MHC haplotype alone is unlikely to explain the dramatic differences that we observed, since BCG produces a large and diverse array of potential antigens. In addition, two strains in our panel (129 and B6) shared the same H2 haplotype and were differentially protected, further indicating that additional mechanisms determine BCG efficacy. Similarly, while the relative ability to eradicate BCG could control efficacy, previous studies found that this vaccine protects animals of widely varying BCG susceptibilities, which were determined by Nramp1 (Slc11a1) polymorphism (44). Instead, the observed correlation between the propensity of an unvaccinated strain to produce IFN-γ after *M. tuberculosis* infection and the degree of BCG-induced protection suggests that intrinsic bias in the antimycobacterial immune response could influence vaccine efficacy. While this correlation was driven by both classical and wild-derived lines, the CAST and PWK lines were particularly strong outliers. These strains made very low levels of IFN-γ after infection and received virtually no benefit from BCG. Thus, it is possible that while BCG is a robust inducer of canonical antimycobacterial Th1 responses, it is unable to stimulate the immune response(s) that protect these highly divergent strains.

The low and variable degree of protection elicited by BCG in natural populations could be due to either a general lack of efficacy or a differential effect in distinct individuals. It is important to understand the relative importance of these two factors, since entirely different approaches are necessary to overcome them. Our observations using a model population of genetically diverse mice indicate that genetic diversity in the host population could be a major factor limiting BCG efficacy. Based on these findings, it is not clear that optimizing a vaccine to protect a single standard laboratory strain of mouse will produce an intervention that is broadly efficacious in an outbred population or even that a single vaccine is capable of protecting genetically diverse individuals. Instead, optimizing a vaccine or set of vaccines to protect nonresponding strains may represent a more effective strategy. Furthermore, understanding the immunological biases that determine TB susceptibility and vaccine efficacy would facilitate the identification of individuals that are genuinely at risk. The large panel of recombinant inbred Collaborative Cross lines that is currently available will allow the genetic dissection of these traits and should facilitate the development of more broadly effective vaccination strategies.

## MATERIALS AND METHODS

### Ethics statement.

The animal studies were approved by the Institutional Animal Care and Use Committee at the University of Massachusetts Medical School (UMMS; Animal Welfare Assurance no. A3306-01), using the recommendations from the *Guide for the Care and Use of Laboratory Animals* of the National Institutes of Health and the Office of Laboratory Animal Welfare (75).

### Mice.

C57BL/6J (stock number 0664), A/J (stock number 0646), 129SvImJ (stock number 02448), NZO/HiLtJ (stock number 02105), NOD/ShiLtJ (stock number 01976), WSB/EiJ (stock number 01145), PWK/PhJ (stock number 3715), and CAST/EiJ (stock number 0928) mice were purchased from the Jackson Laboratory (Bar Harbor, ME, USA), and Collaborative Cross strains CC001, CC002, CC019, and CC042 were purchased from the University of North Carolina (Chapel Hill, NC, USA). Male mice were 8 to 12 weeks old at the start of all experiments. Mice were housed under specific-pathogen-free conditions and in accordance with the University of Massachusetts (UMASS) Medical School IACUC guidelines.

### Vaccination.

Mice were vaccinated with 10^5^ CFU BCG (BCG SSI strain, resuspended in 0.04% phosphate-buffered saline [PBS]–Tween 80) via the subcutaneous route (100 µl per mouse). Mice were rested for 12 weeks; 3 mice per genotype were euthanized at 12 weeks postvaccination and their spleens homogenized and plated to test for BCG persistence. Twenty-four mice per genotype were subsequently infected with *M. tuberculosis*, of which 12 were vaccinated and 12 were naive.

### Experimental infection and bacterial quantification.

Infection with *M. tuberculosis* (H37Rv strain; phthiocerol dimycoserosate [PDIM] positive) was performed via the aerosol route, with mice receiving 50 to 200 CFU. Bacteria were cultured in 7H9 medium containing 0.05% Tween 80 and oleic acid-albumin-dextrose-catalase (OADC) enrichment (Becton, Dickinson). For infections, mycobacteria were suspended in PBS-Tween 80 (0.05%); clumps were dissociated by sonication, and inoculum delivered via the respiratory route using an aerosol generation device (Glas-Col). To determine CFU, mice were anesthetized via inhalation with isoflurane (Piramal) and euthanized via cervical dislocation, the organs aseptically removed and individually homogenized, and viable bacteria enumerated by plating 10-fold serial dilutions of organ homogenates onto 7H10 agar plates. Plates were incubated at 37°C, and *M. tuberculosis* colonies counted after 21 days.

### Cytokine measurement in tissue homogenates.

Murine cytokine concentrations were measured from culture supernatants (prepared from uninfected splenocytes from 3 mice per genotype that were diluted to 1 million cells per well and stimulated by phorbol 12-myristate 13-acetate/ionomycin [PMA/I] or anti-CD3/CD28 antibody for 72 h) or cell-free lung homogenates and quantified using commercial ELISA kits for IFN-γ and TNF (DuoSet, catalog numbers DY485, DY410, and DY402; R & D Systems). The final values are the average values from 3 mice per genotype, with each individual mouse sample run in technical duplicate.

### Light microscopy.

Lung lobes from 3 or 4 individual mice per time point were fixed with 10% neutral buffered formalin, processed, embedded in paraffin, sectioned at 5 µm, and stained with hematoxylin and eosin. Two serial sections were examined by a board-certified veterinary pathologist (G.B.) for qualitative analyses, including assessment of immune/inflammatory cell types, distribution and extent of lesions, presence and absence of necrosis, and extent and content of disrupted lung architecture. The extent of necrosis and neutrophil, macrophage, and lymphocyte influx was then estimated per strain per time point by blindly assessing 10 granulomas chosen at random, and the results reported as the proportional areas occupied by necrosis, neutrophils, lymphocytes, and macrophages.

### Statistical analysis.

All data are represented as mean values and standard deviations (SD). The statistical significance of differences between data groups was determined using the unpaired two-tailed Student’s *t* test, and that of differences in genotypes compared to the standard B6 strain by one-way ANOVA with Tukey’s multiple comparison test, using GraphPad Prism 7. Pearson’s correlation was used to determine correlations between measured traits, and the results visualized using corrplot version 0.77 (ordered by hclust) in R version 3.2.4.

## SUPPLEMENTAL MATERIAL

Figure S1  IFN-γ production *in vitro* from stimulated splenocytes. Spleens were collected from uninfected parental strains and homogenized, and splenocytes were counted, plated, stimulated with either PMA/I or anti-CD3/CD28 antibody, and incubated for 72 h at 37°C before supernatants were collected and IFN-γ was measured by ELISA. Each bar represents the average value from 3 mice, with ELISAs performed in technical duplicate. Error bars show standard deviations. Download Figure S1, TIF file, 10.4 MB

Figure S2  Lung CFU, spleen CFU, and weight change effects from BCG vaccination by individual mouse genotype. Mice were vaccinated with BCG (*n* = 12 per genotype) or left naive (*n* = 12 per genotype) and rested for 12 weeks prior to being infected with *M. tuberculosis*. Mice were weighed and euthanized at 4 and 14 weeks (6 mice per genotype, per treatment condition at each time point), and lung and spleen CFU were enumerated. Protection was calculated by comparing average CFU between naive and BCG-vaccinated groups. Statistical significance was determined by unpaired *t* test (*, *P* < 0.05; **, *P* < 0.01; ***, *P* < 0.001). Download Figure S2, PDF file, 1 MB
