# Supplementary figures and images for: Tuberculosis Susceptibility and Vaccine Protection Are Independently Controlled by Host Genotype
Source: mBio. 2016 Sep 20;7(5):e01516-16. doi: 10.1128/mBio.01516-16 (PMC5030360; doi:10.1128/mBio.01516-16)

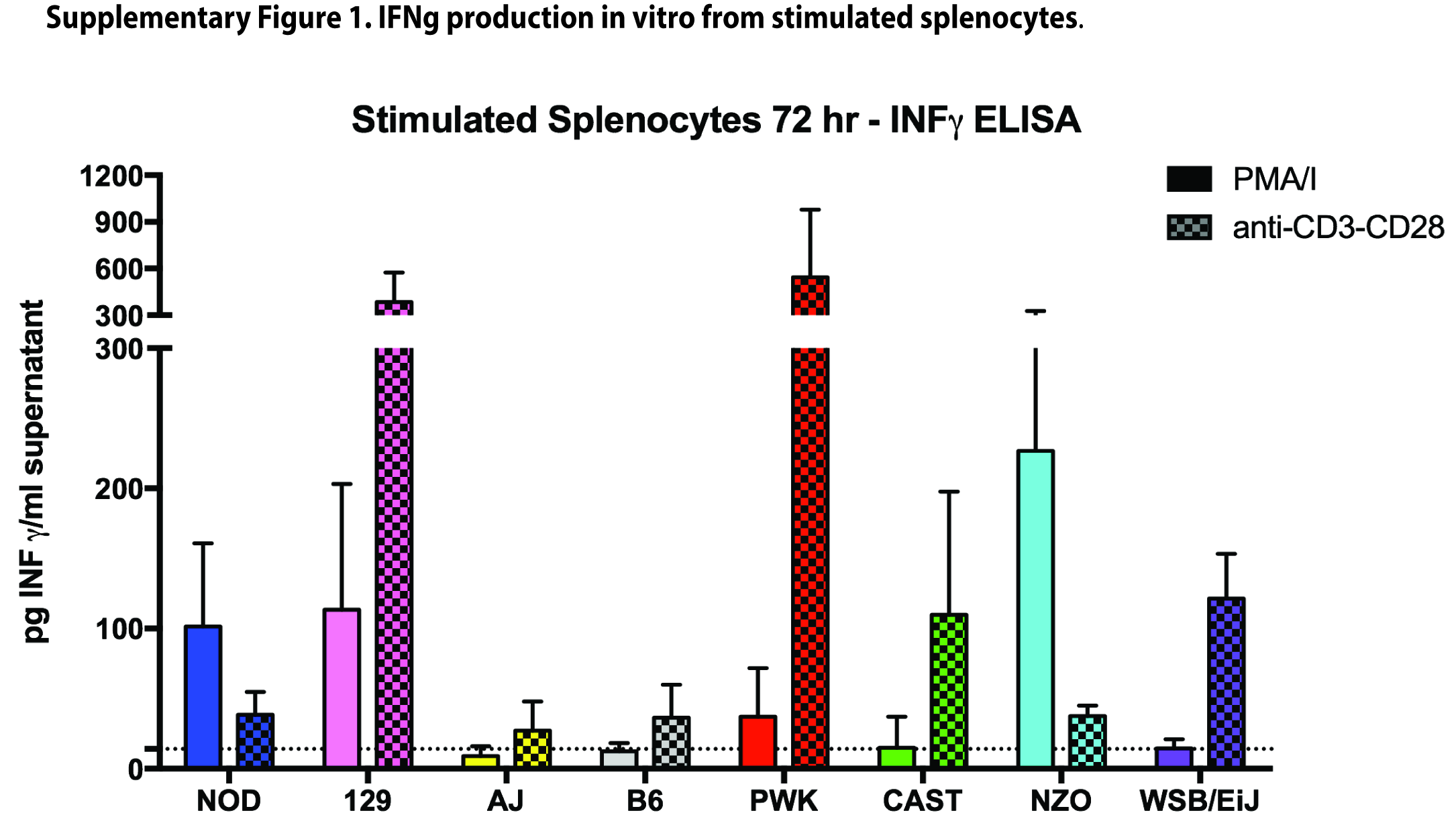

Supplement: Figure S1 — IFN-γ production in vitro from stimulated splenocytes. Spleens were collected from uninfected parental strains and homogenized, and splenocytes were counted, plated, stimulated with either PMA/I or anti-CD3/CD28 antibody, and incubated for 72 h at 37°C before supernatants were collected and IFN-γ was measured by ELISA. Each bar represents the average value from 3 mice, with ELISAs performed in technical duplicate. Error bars show standard deviations. Download [file mbo004162994sf1.tif]
