# Supplementary material for: Tuberculosis Susceptibility and Vaccine Protection Are Independently Controlled by Host Genotype
Source: mBio. 2016 Sep 20;7(5):e01516-16. doi: 10.1128/mBio.01516-16 (PMC5030360; doi:10.1128/mBio.01516-16)

Supplementary Figure 2: Vaccine protection by individual mouse genotype

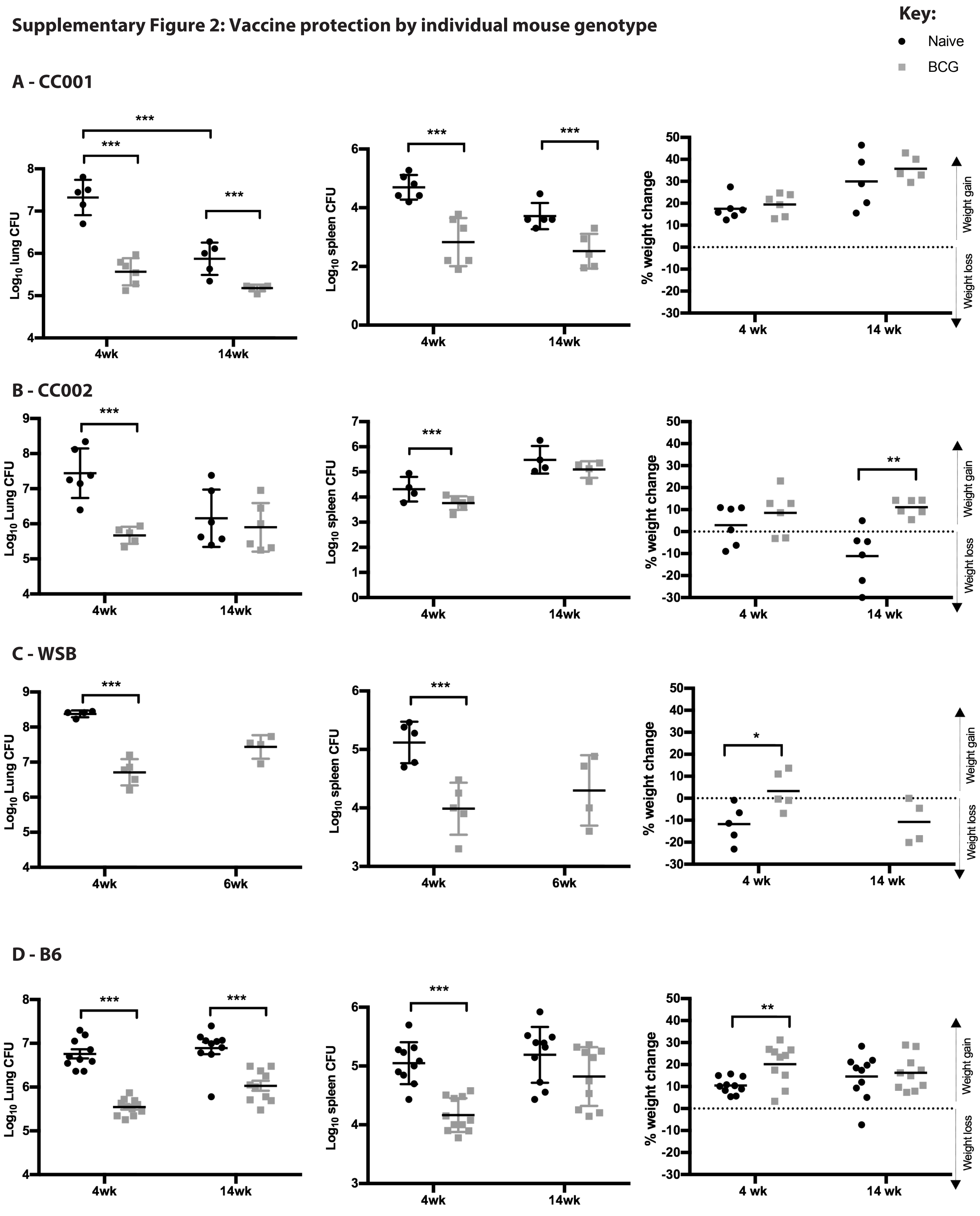

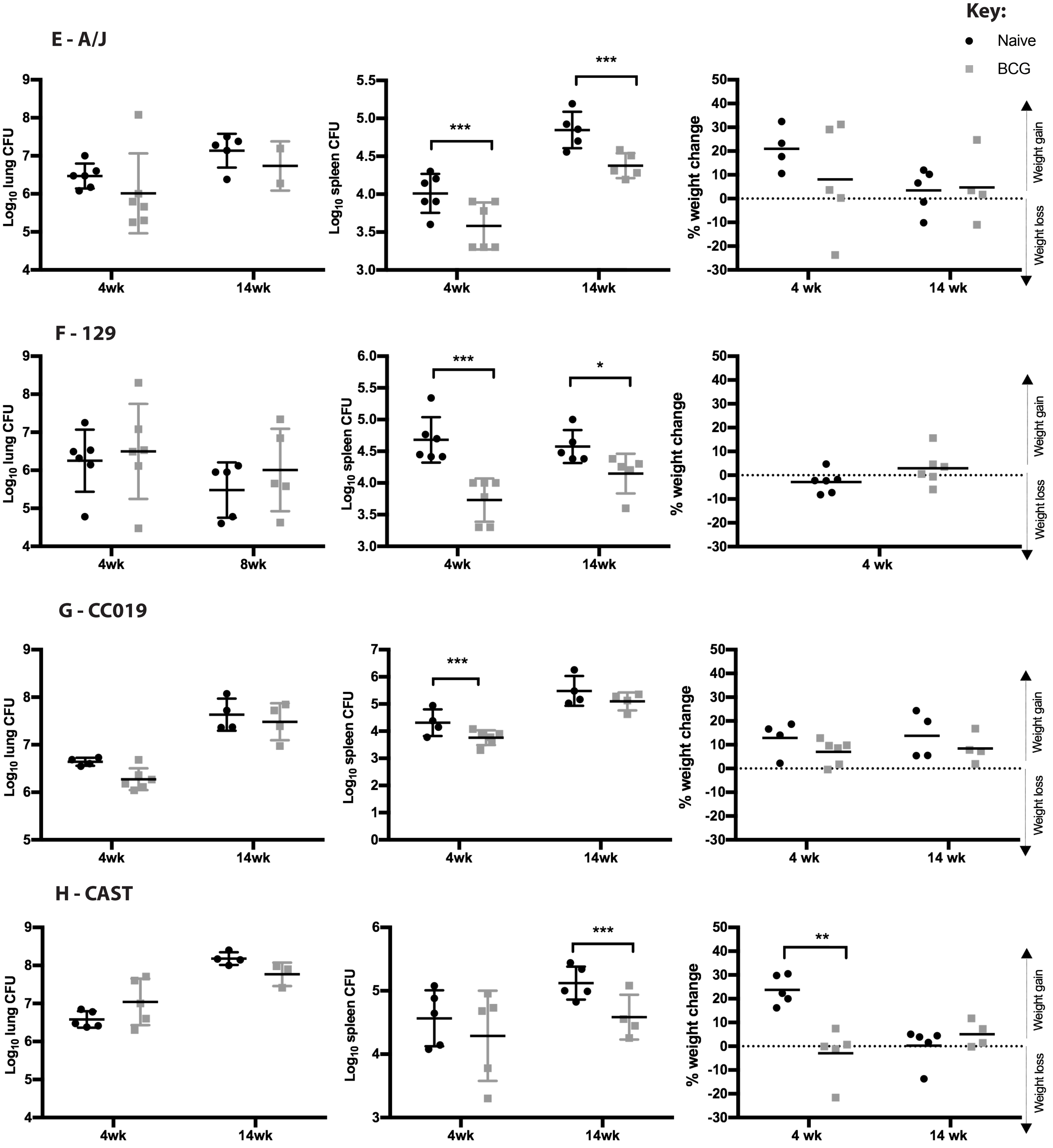
Key:
Naive
BCG
E - A/J
F - 129
G - CC019
H - CAST
Log10 lung CFU
Log10 spleen CFU
% weight change
Weight gain
Weight loss
4wk
8wk
14wk
4 wk
14 wk
***
**
*

Key:
- Naive
- BCG

I - PWK

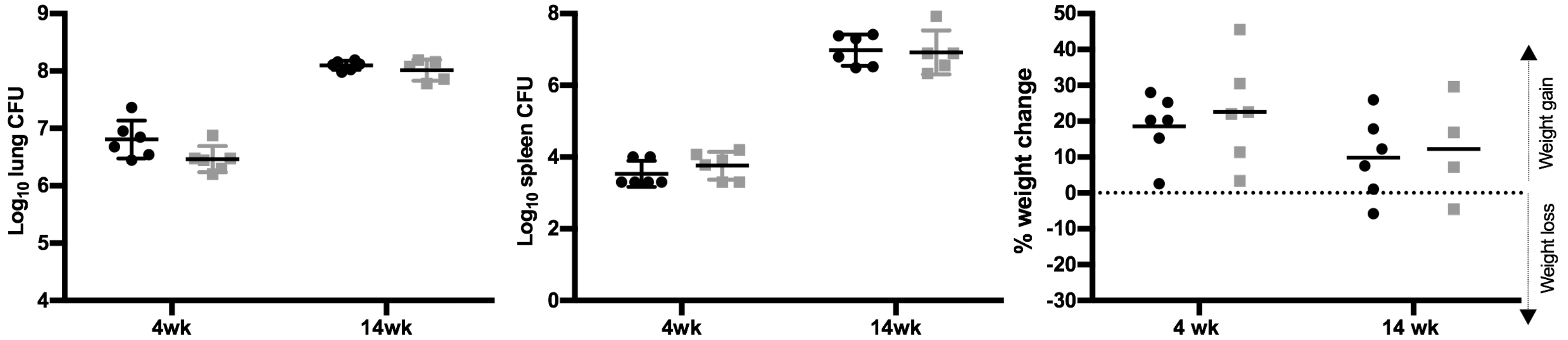

J - NOD

Key:
● Naive
■ BCG

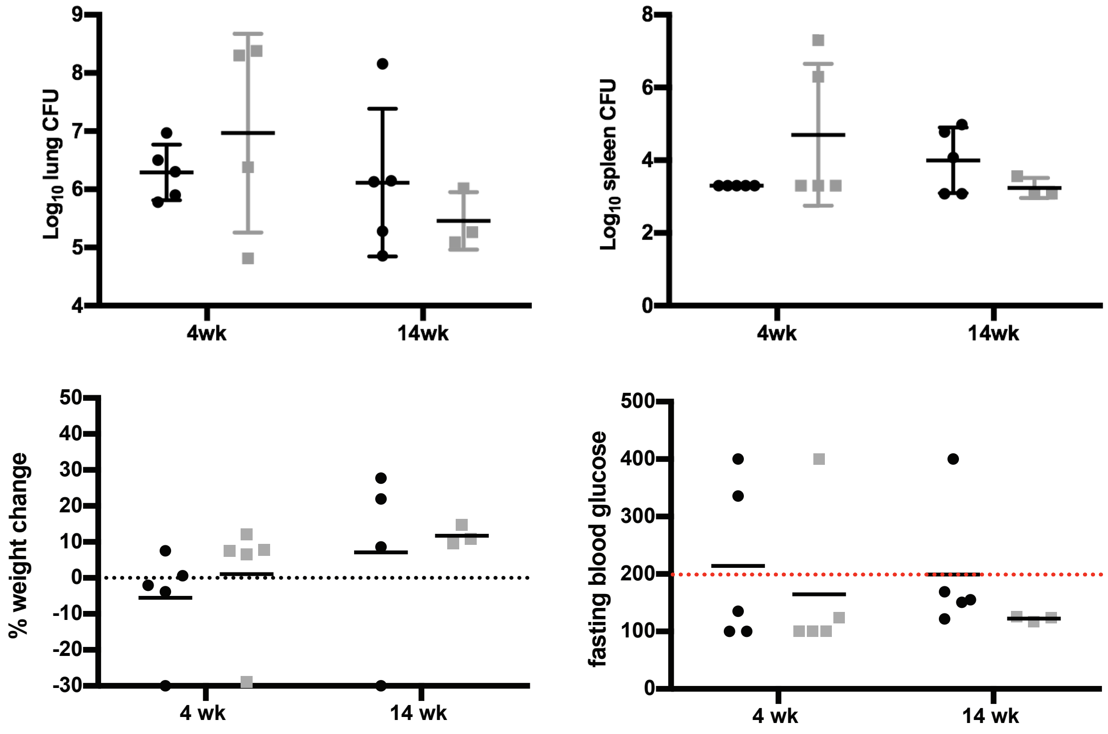

K - NZO

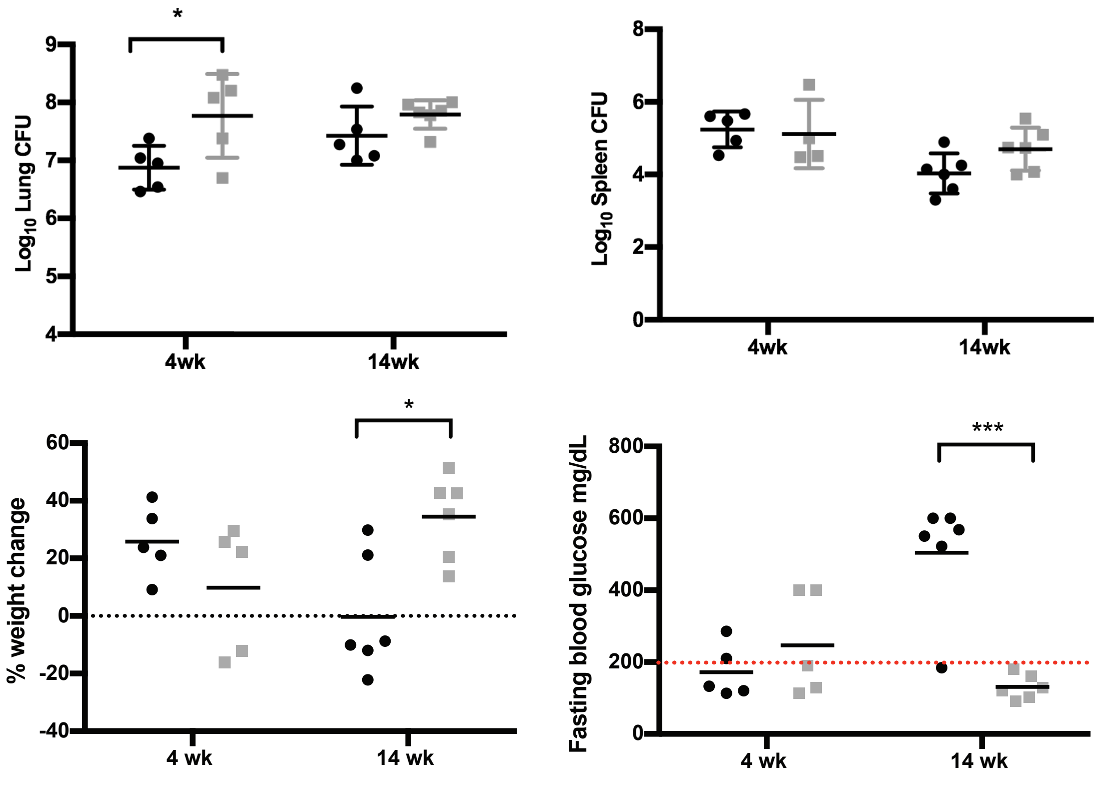

Supplement: Figure S2 — Lung CFU, spleen CFU, and weight change effects from BCG vaccination by individual mouse genotype. Mice were vaccinated with BCG (n = 12 per genotype) or left naive (n = 12 per genotype) and rested for 12 weeks prior to being infected with M. tuberculosis. Mice were weighed and euthanized at 4 and 14 weeks (6 mice per genotype, per treatment condition at each time point), and lung and spleen CFU were enumerated. Protection was calculated by comparing average CFU between naive and BCG-vaccinated groups. Statistical significance was determined by unpaired t test (*, P < 0.05; **, P < 0.01; ***, P < 0.001). Download [file mbo004162994sf2.pdf]
